# A new chelonibiid from the Miocene of Zanzibar (Eastern Africa) sheds light on the evolution of shell architecture in turtle and whale barnacles (Cirripedia: Coronuloidea)

**DOI:** 10.1111/1749-4877.12554

**Published:** 2021-06-05

**Authors:** Alberto COLLARETA, William A. NEWMAN, Giulia BOSIO, Giovanni COLETTI

**Affiliations:** ^1^ Dipartimento di Scienze della Terra Università di Pisa Pisa Italy; ^2^ Museo di Storia Naturale Università di Pisa Calci Italy; ^3^ MBRD Scripps Institution of Oceanography La Jolla California USA; ^4^ Dipartimento di Scienze dell'Ambiente e della Terra Università degli Studi di Milano‐Bicocca Milano Italy

**Keywords:** †*Chelonibia zanzibarensis* sp. nov, epibiosis, evolution, functional morphology, systematics

## Abstract

The fossil history of turtle and whale barnacles (Coronuloidea: Chelonibiidae, Platylepadidae, Coronulidae and †Emersoniidae) is fragmentary and has only been investigated in part. Morphological inferences and molecular phylogenetic analyses on extant specimens suggest that the roots of whale barnacles (Coronulidae) are to be found among the chelonibiid turtle barnacles, but the hard‐part modifications that enabled early coronuloids to attach to the cetacean skin are still largely to be perceived. Here, we reappraise a fossil chelonibiid specimen from the Miocene of insular Tanzania that was previously referred to the living species *Chelonibia caretta*. This largely forgotten specimen is here described as the holotype of the new species †*Chelonibia zanzibarensis*. While similar to *C. caretta*, †*C. zanzibarensis* exhibits obvious external longitudinal parietal canals occurring in‐between external longitudinal parietal septa that abut outwards to form T‐shaped flanges, a character so far regarded as proper of the seemingly more derived Coronulidae and Platylepadidae. Along with these features, the presence of a substrate imprint on the shell exterior indicates that †*C. zanzibarensis* grasped its host's integument in much the same way as coronulids and platylepadids, albeit without the development of macroscopic parietal buttresses and bolsters. Thin section analyses of the inner parietal architecture of some extant and extinct coronuloids conclusively demonstrate that vestiges of comparable external parietal microstructures are present in some living members of Chelonibiidae. This observation strengthens the unity of Coronuloidea while significantly contributing to our understanding of the evolution of the coronuloid shell structure in adapting to a diverse spectrum of hosts.

## Graphical abstract

We reappraise a fossil chelonibiid specimen from the Miocene of insular Tanzania that was previously referred to the living species *Chelonibia caretta*. This largely forgotten specimen is here described as the holotype of the new species †*Chelonibia zanzibarensis*. Microscopical analyses reveal that †*C. zanzibarensis* grasped its host's integument in much the same way as many modern coronulids—an observation that strengthens the unity of the turtle and whale barnacles while shedding some additional light on the evolution of the coronuloid shell structure in adapting to a broad spectrum of hosts.

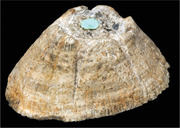

## INTRODUCTION

The sessile barnacles (Crustacea: Cirripedia) included in the superfamily Coronuloidea are known as epibionts of several marine vertebrates (e.g. various species of toothed and baleen whales, sirenians, sea turtles, and other marine reptiles) on one hand and on invertebrates (e.g. gastropods, crabs, and horseshoe crabs) on the other (e.g. Darwin 1854; Gruvel 1905; Pilsbry 1916; Krüger 1940; Newman 1996; Liu & Ren 2007; Buckeridge *et al*. 2018; Dreyer *et al*. 2020). The members of Coronuloidea are currently assigned to 4 families, namely, Coronulidae, Chelonibiidae, Platylepadidae, and the somewhat enigmatic †Emersoniidae (Newman 1996; Collareta & Newman 2020). Whereas the coronulids are obligate commensals of cetaceans, the chelonibiids and platylepadids exhibit more generalist host habits, albeit most species live preferentially or exclusively on the skin, carapace, or plastron of chelonians (e.g. Ross & Newman 1967; Ross & Frick 2011; Hayashi 2013), hence their vernacular name, “turtle barnacles”. The fossil history of Coronuloidea is still fragmentary and only partly investigated; in particular, fossils attributed to the currently monotypic family Chelonibiidae are largely reported as the remains of the extant genus *Chelonibia* Leach, 1817 from various Plio‐Pleistocene deposits worldwide (Collareta *et al*. 2016, and references therein).

The monophyly of Coronuloidea has recently been confirmed by means of molecular phylogeny (e.g. Hayashi *et al*. 2013; Pérez‐Losada *et al*. 2014), which also suggested that the evolutionary roots of coronulids are to be found among the chelonibiids. Nevertheless, morphological characters that might confirm a close relationship between the turtle and the whale barnacles have long been wanting, and the hard‐part modifications that enabled early coronuloids to effectively grasp the cetacean skin have, until now, not been identified. The situation was clear to Charles Darwin, who in his celebrated monograph on the sessile cirripedes (Darwin 1854: pp. 153–154) stated: “I have been greatly tempted to follow Drs. Leach and Gray, who have separated the second of these groups, containing the genera *Coronula*, *Tubicinella*, *Xenobalanus*, and *Platylepas*, into the sub‐family of the Coronulinæ. Certainly these genera have a peculiar aspect in common, and agree in being parasitic and imbedded in the skin of Cetaceans, as is the case with the first three genera, or in that of turtles, manatee, and sea‐snakes, as in *Platylepas*. Though these genera possess several peculiar characters, yet I can find none common to all four, excepting their imbedment in the skin of Vertebrata, their double branchiæ, and their non‐articulated opercular valves […] hence, I repeat, I have not thought it prudent to admit the sub‐family of the Coronulinæ, though in many respects a very natural group”. Some pages further on Darwin (1854: p. 383) had become even more doubtful as he stated: “There is but little special affinity between these genera [i.e*. Chelonibia* and *Coronula* Lamarck, 1802]; and I regret that they have come to be placed one after the other in this work […] the many points of difference, however, in the structure of the shell and of the opercular valves, and especially in the cementing apparatus of the basal membrane, and in the branchiæ, all prove that the genera are very distinct”.

In the present paper, we reappraise a fossil chelonibiid specimen from the Miocene of Pemba Island (Zanzibar archipelago; Tanzania) that was originally described by Withers (1928) as belonging to the living species *Chelonibia caretta* (Spengler, 1790). While displaying all the diagnostic hard‐part characters of living chelonibiids, and even an indisputable resemblance to the extant species *C. caretta*, this largely forgotten specimen also exhibits large, obvious, external longitudinal parietal canals, so far thought to represent a derived character of Coronulidae and Platylepadidae (see e.g. Ross & Frick 2011). Along with these canals, the presence of a distinct substrate imprint on the shell exterior strongly suggests that this chelonibiid penetrated its host's tissues with a grasping style that recalls that of extant coronulids as well as platylepadids. Therefore, we undertook a thin section analysis of the inner parietal architecture of some extant and extinct coronuloids which proved that vestiges of external longitudinal parietal canals are present in some living forms of *Chelonibia*—an observation that strengthens the unity of the turtle and whale barnacles while shedding some additional light on the evolution of the coronuloid shell structure in adapting to (and spreading on) an amazingly diverse spectrum of hosts. Herein, we describe a new extinct species of *Chelonibia* on the basis of Withers’ specimen. Furthermore, we elucidate the significance of Withers’ specimen for our understanding of the coronuloid *bauplan* and its early evolution as deduced from our thin section analysis.

## MATERIALS AND METHODS

### Geological setting

Following the drifting of Madagascar from mainland Africa during the Triassic and Jurassic, a passive margin developed along the eastern coast of Central Africa (Nicholas *et al*. 2007). This margin was characterized by remarkable tectonic stability from the late Cretaceous to the early Oligocene (Nicholas *et al*. 2007). Subsequently, the region was affected by extensional tectonics related to the East African Rift, which led to the opening of several basins in the coastal and offshore areas of what is now Tanzania (Nicholas *et al*. 2007). These basins are mainly filled by fluvio‐deltaic and shallow‐marine deposits ranging in age from the Oligocene to the Miocene (Stockley 1942; Eames & Kent 1955; Stewart *et al*. 2004; Nicholas *et al*. 2007; Harzhauser 2009). These sedimentary deposits constitute the bulk of the Zanzibar archipelago, which includes Unguja (also known as Zanzibar Island), Pemba, and Mafia, along with many other smaller islands.

**Figure 1:**
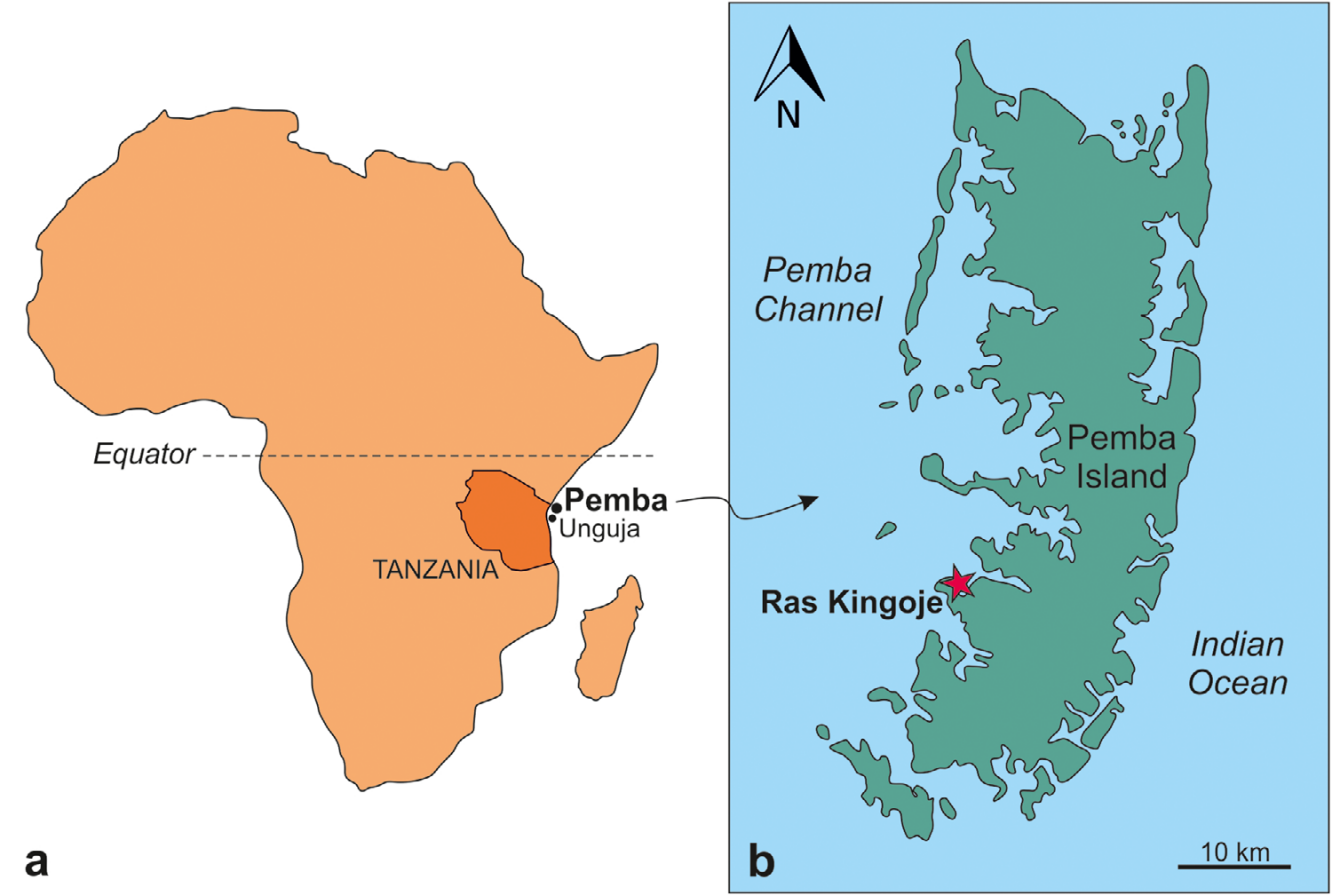
Geographical setting. (a) Location of the Unguja and Pemba islands in the Zanzibar Archipelago (Tanzania, Eastern Africa). (b) Location of Ras Kingoje, the type locality of †*Chelonibia zanzibarensis* sp. nov., on Pemba Island.

The Miocene succession of Pemba Island (Fig. 1) comprises 2 main units: the Chake‐Chake beds and the overlying Weti beds (Stockley 1942; Pickford 2008). Whereas the Weti beds are mostly unfossiliferous, the Chake‐Chake beds are characterized by a rich fossil assemblage that also includes the chelonibiid specimen described by Withers (1928) and re‐evaluated in the present work. The diverse large benthic foraminiferal assemblage of the Chake‐Chake beds also includes age‐diagnostic taxa such as †*Miogypsina* Sacco, 1893 and †*Nephrolepidina martini* Schlumberger, 1900 (Stockley 1942). According to Van Vessem (1978), the latter characterizes the Serravallian stage of the Miocene. However, more recent analyses on the central Indian Ocean record highlighted that, by middle to late Serravallian times, this species was being replaced by taxa closer to †*Nephrolepidina rutteni* (van der Vlerk, 1924) (Coletti *et al*. 2018b). On the other hand, an early Miocene (Aquitanian‐Burdigalian) age has been proposed by BouDagher‐Fadel (2018) for the Chake‐Chake beds also based on large benthic foraminiferal assemblages. Finally, the abundant terrestrial vertebrate remains that locally characterize the Miocene succession of Pemba Island exhibit striking similarities with the earliest middle Miocene faunas of the African continent (Pickford 2008). Given these considerations, and taking into account that the accurate stratigraphic position of Withers’ specimen within the Chake‐Chake succession is unfortunately unknown, its geological age should be regarded as between the early and the middle Miocene.

### Systematics and anatomical terminology

**Figure 2:**
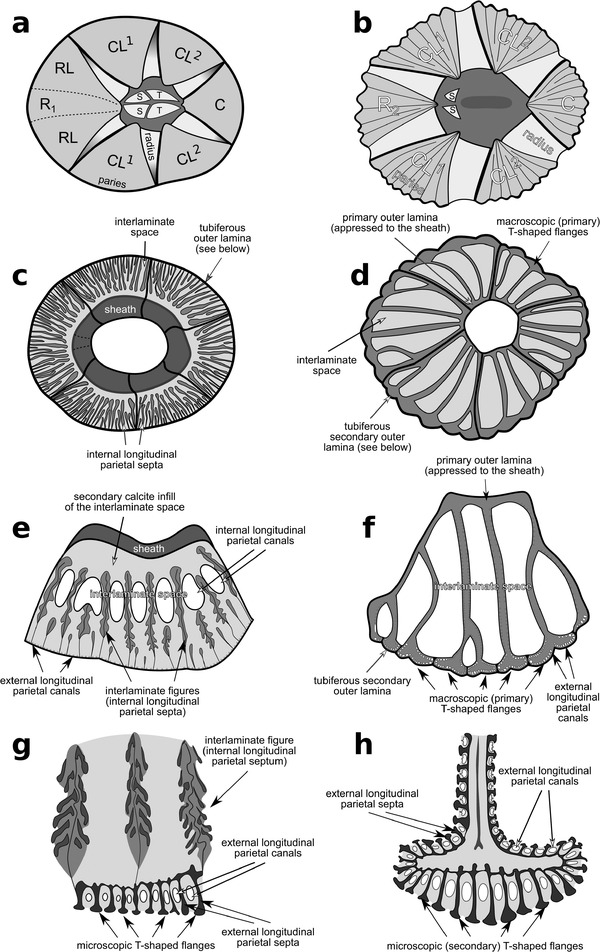
Summary of the nomenclatural scheme embraced in the present paper for referring to the macroscopic and microscopic features of the coronuloid shells. Panels (a), (c), (e), and (g) depict the shell of a generalized chelonibiine (*sensu* Harzhauser *et al*. 2011, and the present work); panels (b), (d), (f), and (h) depict the shell of a generalized coronuline (*sensu* Ross & Frick 2011). (a,b) Apical view of the shell, with the main structures and districts labeled. (c,d) Basal view of the shell (note that the barnacle's soft tissues and opercular plates are not shown). (e,f) Transverse section at mid‐height through a compartment paries, with the main structures and districts labeled. (g,h) Detail of the outer wall in transverse section (note that the T‐shaped flanges and related external longitudinal parietal canals of chelonibiids are described for the first time in the present work). Abbreviations: C, carina; CL^1,^ carinolateral^1^; CL^2^, carinolateral^2^; R_1_, rostrum; R_2_, compound rostrum (*sensu* Harzhauser *et al*. 2011); RL, rostrolateral; S, scutum; T, tergum.

Various nomenclatural schemes have encompassed the complex shell structures of coronuloid barnacles. The anatomical terminology followed herein derives mainly from several works by Darwin (1854), Pilsbry (1916), Ross and Newman (1967), Davis (1972), Newman and Ross (1976), Buckeridge (1983), Harzhauser *et al*. (2011), Ross and Frick (2011), Collareta *et al*. (2016), Collareta (2020), and Collareta and Newman (2020). The nomenclatural scheme utilized in the present paper for referring to the macroscopic and microscopic features of the coronuloid shell is indicated in Fig. 2.

Following the classification scheme proposed by Newman (1996), the superfamily Coronuloidea is regarded herein as comprising 4 families, namely, Chelonibiidae, Coronulidae, †Emersoniidae, and Platylepadidae. We must mention, however, that a just‐published article by Chan *et al*. (2021) proposes to redefine the content of Coronuloidea based on molecular patterns. Thus, following Chan *et al*. (2021), Coronuloidea should also include the austrobalanids, bathylasmatids, and tetraclitids, with the coronulids, emersoniids, and platylepadids being merged into a single family, Coronulidae. In the same paper, an attribution of *Stephanolepas* Fischer, 1886 to the subfamily Chelonibiinae is also provided (Chan *et al*. 2021). As somewhat anticipated by Collareta *et al*. (2020), we mostly disagree with such an arrangement of the coronuloids, which proposes drastic changes that should also be properly substantiated by extensive morphological observations on extant and extinct forms. Thus, while acknowledging the impressive amount of results and interpretations provided by Chan *et al*. (2021), and pending a detailed reappraisal of the morphological affinities of the turtle and whale barnacles, we prefer to retain the classification scheme proposed by Newman (1996) for the moment being.

### Institutional abbreviations

KBIN, Koninklijk Belgisch Instituut voor Natuurwetenschappen (Belgium); NHMUK, The Natural History Museum, London (United Kingdom); MCSNC, Museo Civico di Storia Naturale di Comiso (Italy); MSNV, Museo di Storia Naturale di Venezia (Italy); MSNUP, Museo di Storia Naturale, Università di Pisa (Italy); MZUF, Museo di Storia Naturale, Sezione di Zoologia “La Specola”, Università degli Studi di Firenze (Italy); SAM‐PK, Natural History Iziko South African Museum (South Africa).

### Comparisons and thin section analysis

Recent and fossil shells of Chelonibiidae, Coronulidae, and Platylepadidae from the KBIN, MCSNC, MSNV, MSNUP, MZUF, and SAM‐PK collections were directly examined for comparison purposes.

Specimens of the extinct and extant coronuloid species *Chelonibia caretta*, *Chelonibia testudinaria* (Linnaeus, 1758) (*testudinaria* morph), *Chelonibia testudinaria* (*patula* morph), †*Coronula bifida* Bronn, 1831, and *Coronula diadema* (Linnaeus, 1767) were prepared as thin sections in order to observe their parietal microstructures. Each specimen was embedded in epoxy resin and then cut with a rock saw at Milano‐Bicocca University. The resulting surface was polished with abrasive powder and then glued to a standard petrographic glass slide. The excess material was cut away with a rock saw and then the specimen was ground to a thickness of 30–40 μm. All the sections were analyzed under a Leitz Orthoplan transmitted light microscope (OM) at Pisa University at magnifications up to 160×.

## SYSTEMATICS

Subclass Cirripedia Burmeister, 1834

Superorder Thoracica Darwin, 1854

Order Balanomorpha Pilsbry, 1916 (=Order Sessilia Lamarck, 1818, *sensu* Buckeridge & Newman, 2006)

Infraorder Neobalanoformes Gale (*sensu* Kočí *et al*. 2017, to accommodate Neobalanomorpha Gale in Gale & Sørensen, 2015)

Superfamily Coronuloidea Leach, 1817

### Emended diagnosis (modified after Ross & Frick 2011)

Neobalanoformes with operculum occupying substantially less than whole orifice area; scutum and tergum ranging from weakly developed and articulated to disarticulated, variously reduced or completely absent; wall either eight‐plated (rostrum‐rostrolatera‐carinolatera^1^‐carinolatera^2^‐carina) or six‐plated (compound rostrum‐carinolatera^1^‐carinolatera^2^‐carina), parietes with internal and/or external longitudinal parietal canals; wherever present, external longitudinal parietal canals defined in‐between T‐shaped flanges that abut from external longitudinal parietal septa, either macroscopic or microscopic and sometimes vestigial; basis membranous.

### Included families

Chelonibiidae Pilsbry, 1916; Coronulidae Leach, 1817; †Emersoniidae Ross in Ross & Newman, 1967; Platylepadidae Newman & Ross, 1976.

Family Chelonibiidae Pilsbry, 1916

### Included subfamilies

Chelonibiinae Pilsbry, 1916; †Protochelonibiinae Harzhauser & Newman in Harzhauser *et al*., 2011 (see also Table 1).

**Table 1:** Updated classification of the chelonibiids (family Chelonibiidae), with indication of the chronostratigraphic range of each specific and supraspecific taxon

Family Chelonibiidae Pilsbry, 1916: 262 (nom. transl. Newman, 1996 [*ex* Chelonibiinae Pilsbry, 1916: 262]), L. Olig.–Rec.
Subfamily †Protochelonibiinae Harzhauser & Newman, 2011 (*in* Harzhauser, Newman & Grunert, 2011: 474), L. Olig.– U. Plio.
†*Protochelonibia* Harzhauser & Newman, 2011 (*in* Harzhauser, Newman & Grunert, 2011: 474), L. Olig.– U. Plio.
†*Protochelonibia submersa* Harzhauser & Newman, 2011 (*in* Harzhauser, Newman & Grunert, 2011: 475), L. Mio., Austria and Italy.
†*Protochelonibia capellinii* (De Alessandri, 1895: 300), ?U. Plio., Italy.
†*Protochelonibia melleni* (Zullo, 1982: 3), L. Olig., Mississippi (USA).
Subfamily Chelonibiinae Pilsbry, 1916: 262.
*Chelonibia* Leach, 1817: 68, L.‐M. Mio–Rec.
{“*Chelonibia caretta* species‐group”, informal grouping herein, L.‐M. Mio–Rec.}
*Chelonibia caretta* (Spengler, 1790: 185), L.‐M. Mio–Rec.
†*Chelonibia zanzibarensis* Collareta & Newman, sp. nov., for “*C. caretta*” of Withers (1928: 391), L.‐M. Mio, Zanzibar (Tanzania).
{“*Chelonibia testudinaria* species‐group”, informal grouping herein, L. Mio–Rec.}
*Chelonibia testudinaria* (Linnaeus, 1758: 668), and the following morphs, as indicated by the prefix alpha (a).
a*Chelonibia patula* (Ranzani, 1817: 86), here regarded as *C. testudinaria* (*patula* morph), Plio–Rec.
a*Chelonibia manati* Gruvel, 1903: 116, here regarded as *C. testudinaria* (*manati* morph), Rec.
a*Chelonibia ramosa* Korschelt, 1933: 2, here regarded as *C. testudinaria* (*ramosa* morph), Rec.
a*Chelonibia manati crenatibasis* Pilsbry, 1916: 266, here regarded as not unambiguously distinguishable from the *manati* morph above, Rec.
a*Chelonibia manati lobatobasis* Pilsbry, 1916: 266, here regarded as not unambiguously distinguishable from the *manati* morph above, Rec.
a*Chelonibia patula dentata* Henry, 1943: 370, here regarded as not unambiguously distinguishable from the *patula* morph above, Rec.
†*Chelonibia solida* Withers, 1929: 568, stat. nov., herein, L. Mio., France.
†*Chelonibia hemisphaerica* Rothpletz & Simonelli, 1890: 724, Plio., Canaries (Spain).

L, Lower; M., Middle; Mio., Miocene; Olig., Oligocene; Plio., Pliocene; Rec., Recent; U, Upper.

### Diagnosis

See Harzhauser *et al*. (2011).

Subfamily Chelonibiinae Pilsbry, 1916

### Type and only included genus

*Chelonibia* Leach, 1817.

### Diagnosis

See Harzhauser *et al*. (2011).

Genus *Chelonibia* Leach, 1817

### Type species

*Lepas testudinaria* Linnaeus, 1758.

### Included species

*Chelonibia caretta* (Spengler, 1790); †*Chelonibia hemisphaerica* Rothpletz & Simonelli, 1890; †*Chelonibia solida* Withers, 1929; *Chelonibia testudinaria* (Linnaeus, 1758); †*Chelonibia zanzibarensis* Collareta & Newman, sp. nov. (see also Table 1).

†*Chelonibia zanzibarensis* Collareta & Newman, sp. nov.

Figures 3 and 4

**Figure 3:**
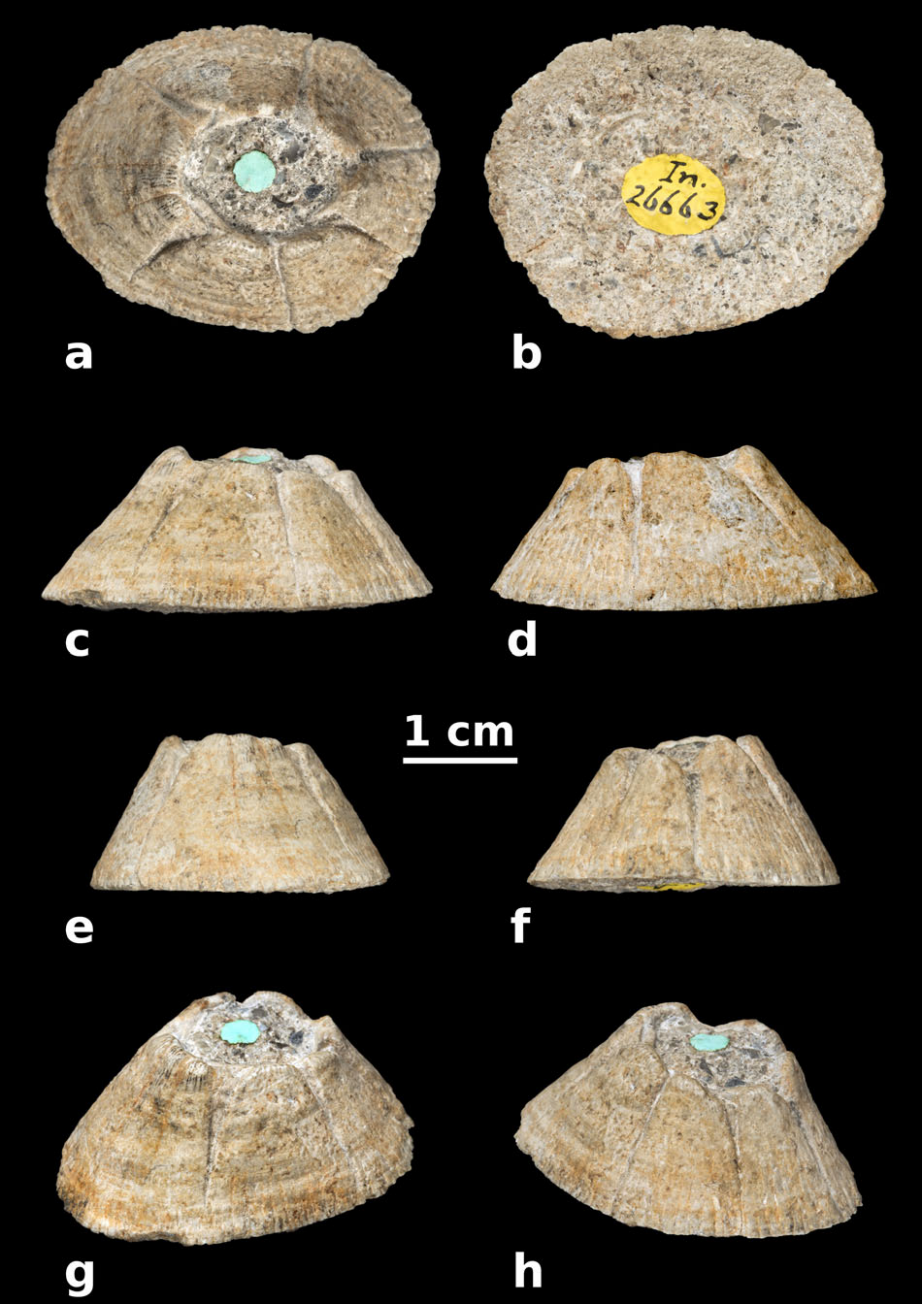
NHMUK PI In.26663, holotype and only known specimen of †*Chelonibia zanzibarensis* sp. nov., complete shell lacking the opercula collected at Ras Kingoje (Pemba Island, Zanzibar archipelago, Indian Ocean) from the Miocene Chake‐Chake beds. (a) Apical view. (b) Basal view. (c) Right lateral view. (d) Left lateral view. (e) Rostral view. (f) Carinal view. (g) Right apicorostrolateral view. (h) Right apicocarinolateral view.

**Figure 4:**
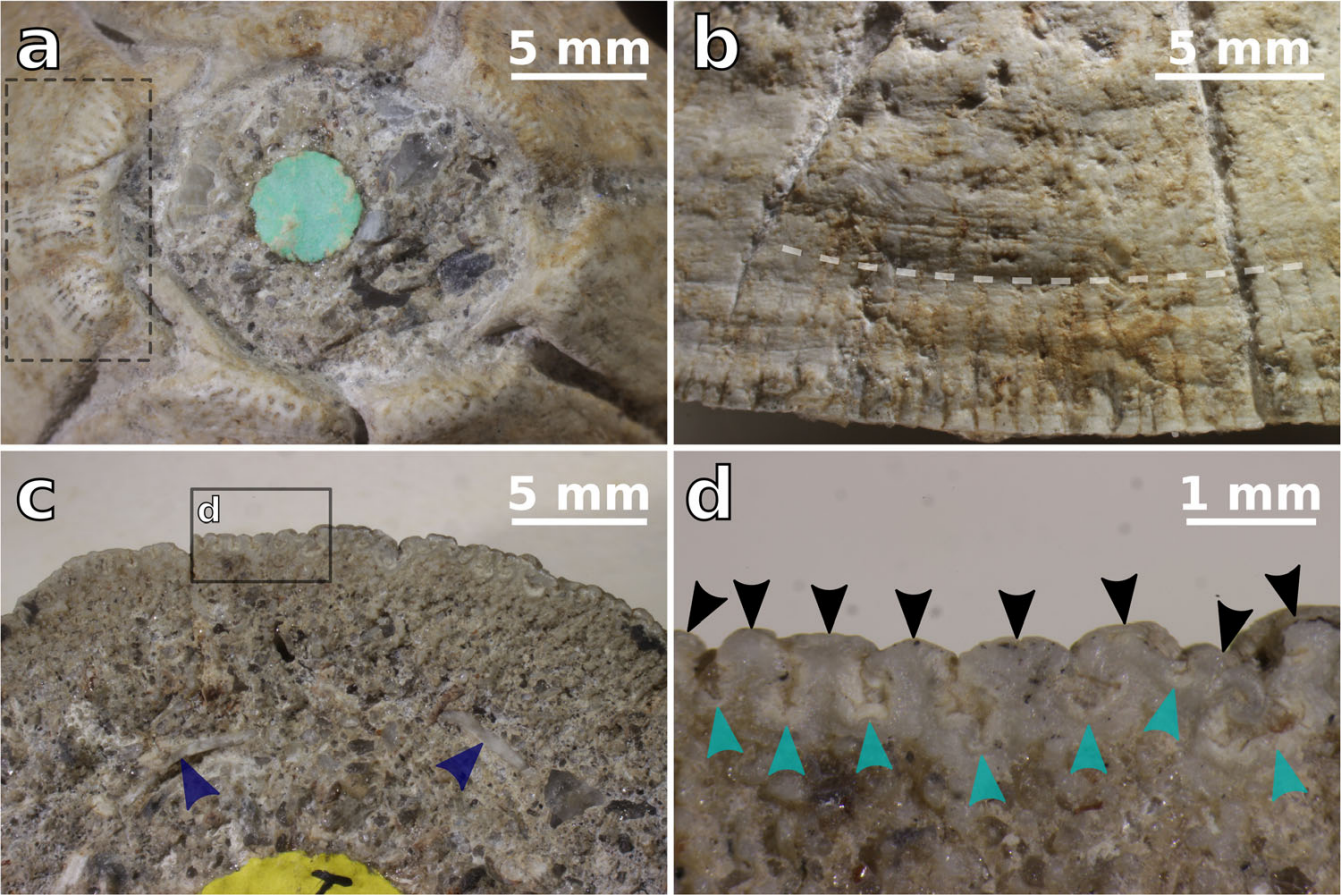
NHMUK PI In.26663, holotype and only known specimen of †*Chelonibia zanzibarensis* sp. nov., complete shell collected at Ras Kingoje (Pemba Island, Zanzibar archipelago, Indian Ocean) from the Miocene Chake‐Chake beds. (a) Close‐up view of the orifice, in apical view; the dashed rectangle indicates the apical portion of the rostral complex—note the large, obvious, external longitudinal parietal canals and septa (some of which bifurcate downwards, thus originating new canals in‐between them). (b) External view of the right CL^1^, displaying a distinct subhorizontal substrate imprint, marked by a dashed line. (c) Close‐up view of the left carinolatera, in basal view—note the depending basal edge of the sheath, indicated by blue arrowheads. (d) Detail of panel (c), showing the presence of external longitudinal parietal canals (indicated by turquoise arrowheads) and related T‐shaped flanges (indicated by black arrowheads) at the periphery of the left CL^1^—note the incipient bifurcation of a T‐shaped flange to originate a new canal on the right side of the panel.

*Chelonibia caretta* (Spengler): Withers 1928, p. 391, text‐fig. at p. 392.

### Short differential diagnosis

A member of *Chelonibia* distinguishable from other congeneric species by the presence of obvious external longitudinal parietal septa that abut to form T‐shaped flanges defining external longitudinal parietal canals in‐between them.

### Extended diagnosis

Shell truncated‐conical; orifice bluntly toothed; parietes thick, broad, sub‐trapezoidal; sutures between rostrum and rostrolaterals rather deeply excavated, especially close to the top of the shell; radii triangular, sunken, unadorned, poorly developed, more so on carinolateral side of rostrolaterals; external longitudinal parietal canals obvious, large, and regularly arranged, bifurcating downwards and separated by external longitudinal parietal septa that abut to form T‐shaped flanges; basal outline oval, distinctly asymmetrical.

### Holotype

An articulated shell, lacking the opercula, currently kept at the Natural History Museum of London under accession number NHMUK PI In.26663.

### Etymology

The species is named after Zanzibar, the Tanzanian archipelago where the holotype was collected.

### Type locality

Ras Kingoje (≈5°17′ S; 39°39′ E), Pemba Island, Zanzibar Archipelago, Tanzania (Fig. 1). Withers (1928) reported NHMUK PI In.26663 as coming from (Stockley's?) site P.68.

### Type horizon

Lower‐Middle Miocene Chake‐Chake beds (see above for more details).

### Holotype dimensions

Carino‐rostral diameter at the shell base—35.8 mm; transverse width of the shell—28.4 mm; shell height at the rostral end—14.1 mm (after Withers 1928). The shell exhibits its maximum height at its rostral end.

### Description and comparisons

NHMUK PI In.26663 displays a high, truncated‐conical profile (Fig. 3c). The shell consists of 8 plates, including the rostrum (R), the left and right rostrolatera (RL), the left and right carinolatera^1^ (CL^1^), the left and right carinolatera^2^ (CL^2^), and the carina (C) (Fig. 3a). As in all the chelonibiines, of the aforementioned plates, the rostrum and rostrolatera are neither fully separable nor completely concrescent, being indeed partly fused to each other to form a tripartite rostral complex (RL‐R‐RL). The paries of R is not acutely triangular as in †*Protochelonibia* Harzhauser & Newman in Harzhauser *et al*., 2011, but rather high and trapezoidal (Fig. 3a), and overall similar to those of the adjacent RLs. The rostral complex is asymmetrical, the left RL being transversely broader than its right antimere (Fig. 3a). The sutures between R and RLs are rather deeply excavated, especially close to the top of the shell, and they tend to fade downwards, but the suture between R and the left RL is well recognizable along the whole height of the shell (Figs 3e and 4a). The apices of the parietes of R and RLs are abraded and consequently blunt, so that the alae of R are partly exposed beneath the adjacent RLs (Fig. 4a). In the upper portion of the shell, a narrow, triangular, sunken radius is observed on the carinolateral side of each RL (Fig. 3a). The CLs and C are broad trapezoidal plates (Fig. 3c,d); they are generally gently convex, but the left CL^2^ is even concavo‐convex (Fig. 3a). The CLs^1^ are transversely broader than the CLs^2^ and C (Fig. 3a,c,d,f). The left CLs are slightly broader transversely than their right antimeres, so that C appears as distinctly displaced rightwards with respect to R (Fig. 3a). The radii are almost nonexistent on CLs. The apices of CLs and C are moderately rounded by erosion, thus providing the shell orifice with a somewhat bluntly toothed aspect. The body chamber is almost completely filled by a hardened volume of the host rock that could not be removed (Figs 3a and 4a). The external surface of the shell has a rough, chalky appearance that is reminiscent of that of many extant specimens of *Chelonibia caretta*. In the upper portion of the shell, and especially in the apical quarter of R and RLs, obvious dark‐colored striations run longitudinally along the substantially smooth external surface of the compartment parietes (Figs 3a and 4a). These striations define external parietal canals that were exposed by sub‐apical erosion of the shell and appear as partly filled by a blackish, possibly carbonaceous material, as well as by diagenetic cement. The external longitudinal parietal canals of NHMUK PI In.26663 are similar to (but distinctly larger than) those observed in various species of *Platylepas* Gray, 1825—for example, *Platylepas hexastylos* (Fabricius, 1798) or †*Platylepas mediterranea* Collareta *et al*., 2019—and are divided from each other by external longitudinal parietal septa. On R, some septa are observed to bifurcate downwards due to the appearance of a new canal (Fig. 4a), so that the interseptal distance does not increase significantly as the paries moderately flares downwards. Since the median and lower portions of the shell are not as abraded as the upper one, here the external longitudinal parietal canals are not visible through the largely unworn external surface of the parietes, which in turn features distinct longitudinal riblets that slightly increase in relief and occasionally bifurcate towards the shell periphery (Figs 3c,d,g,h and 4b). Locally, for example, on the left CL^1^, a shallow but distinct subhorizontal trough is observed at about one‐third of the shell height (measured from the basal margin) (Figs 3c,d,g,h and 4b). Based on the observation of similar features in *C. caretta*, a relatively deep‐penetrating extant species (Darwin 1854; Pilsbry 1916), this trough is here interpreted as some kind of substrate imprint. The basal aspect of the shell is largely hidden by the coarse matrix (Fig. 3b), thus frustrating any attempt to reconstruct the location of soft tissue structures such as the ovarian chambers, whose position within the barnacle shell varies across Coronuloidea (Darwin 1854). Nevertheless, broken portions of the depending basal edge of the sheath are locally observable (Figs 3b and 4c). Radiating internal parietal septa run between the freely projecting lower edge of the sheath and the outer wall to form what was acutely described by Withers (1928) as a wide, flat, calcareous surface (Fig. 4c). As the basal edges of the internal parietal septa are finely denticulated by rounded depending points, they are often difficult to distinguish from the clear‐colored mineral fragments that abundantly occur in the entombing matrix. When viewed basally, the outer wall is thickened and has several irregular T‐shaped flanges that abut outwards, thus defining external longitudinal parietal canals in‐between them (note that these canals are now completely filled by cement) (Fig. 4d). The T‐shaped flanges that are observed at the periphery of the shell correspond to the riblets ornamenting the largely pristine lower portion of the shell (Figs 3c,d,g,h and 4b); in addition, the canals that are seen in basal view correlate with those that are seen through the worn out upper portion of the compartment parietes (Figs 3a and 4a). In some places along the shell periphery, the incipient bifurcation of a T‐shaped flange to originate a new canal is observed (Fig. 4d), hinting at the branching of the external longitudinal parietal canals in the upper part of the shell, as well as at the bifurcation of the riblets in the lower portion of the compartment parietes.

### Remarks

As highlighted elsewhere (e.g. Collareta & Newman 2020), the fossil record of Coronuloidea is still largely fragmentary, and the description herein of the new species †*Chelonibia zanzibarensis*—one of the oldest representatives of the subfamily Chelonibiinae known to date—represents a significant addition to the diversity of extant and extinct chelonibiids. An updated classification of the chelonibiids, stemming from the present study as well as from the recent works by Harzhauser *et al*. (2011), Cheang *et al*. (2013), Zardus *et al*. (2014), and Collareta and Newman (2020), is proposed in Table 1.

Harzhauser *et al*. (2011) were the first to suggest that extant chelonibiids are representative of two different species‐groups, or even distinct genera (but see also Zullo 1982 for a classification of the nominal species, subspecies and varieties of *Chelonibia* into different “facies”). Later, by means of genetic analyses, Cheang *et al*. (2013) and Zardus *et al*. (2014) demonstrated that, out of 4 living species of *Chelonibia*, 3—that is, *Chelonibia testudinaria*, *Chelonibia manati* Gruvel, 1903, and *Chelonibia patula* (Ranzani, 1817)—are synonyms, representing indeed different morphs or ecotypes of the same species (i.e. *C. testudinaria* according to the principle of priority). Moreover, Zardus *et al*. (2014) observed that complemental males are present in all the aforementioned morphs of *C. testudinaria*, an androdioecious sexual mode that contrasts with the typical cirripedian hermaphroditism thought to be present in *Chelonibia caretta* (see also Collareta 2020 in this respect). Besides this prime difference in life history strategies, *C. testudinaria* differs from *C. caretta* on the basis of various hard‐part characters that can be observed in the few chelonibiid fossils known to date. Considering that some *testudinaria*‐like extinct species—that is, †*Chelonibia hemisphaerica* Rothpletz & Simonelli, 1890; †*Chelonibia solida* Withers, 1929 stat. nov.; *C*. “*patula*”—are known from the Miocene and Pliocene (Rothpletz & Simonelli 1890; Withers 1929; Ross 1963), two distinct species‐groups (namely, that of *C. testudinaria* and that of *C. caretta*) seem to have existed through most of the Neogene and Quaternary (Table 1). As regards †*C. zanzibarensis*, our macroscopic morphological investigations show that this extinct species shares with the extant species *C. caretta* a number of skeletal characters that allow for distinguishing it from *C. testudinaria*, namely: (i) a high shell profile; (ii) a distinctly asymmetrical disposition of the rostral compartments; (iii) a carinorostral diameter not greater than ≈50 mm; (iv) a rough and somewhat chalky aspect of the external shell surface; (v) bluntly worn apices of the compartment parietes; (vi) thick parietes that are transversely broad and sub‐trapezoidal in shape; and (vii) diminutive radii that are best developed on the carinolateral side of rostrolaterals and whose external surface is substantially featureless. On the whole, these characters clearly distinguish †*C. zanzibarensis* and *C. caretta* on one hand from *C. testudinaria* and allied forms on the other. Therefore, our reappraisal of the Miocene Tanzanian chelonibiid described by Withers (1928) as belonging to *C. caretta* demonstrates that at least one extinct species of *caretta‐*like chelonibiid existed as early as the early or middle Miocene.

The observation of external longitudinal parietal septa that abut to form T‐shaped flanges defining external longitudinal parietal canals in‐between them comes as some surprise in †*C. zanzibarensis*. Indeed, these kind of complex structures have never been described from any member of Chelonibiidae, whose outer wall has long been regarded as solid, whereas external longitudinal parietal canals and related T‐shaped flanges are known from the other living families of coronuloids, that is, the coronulids and platylepadids (e.g. Darwin 1854; Pilsbry 1916; Newman & Ross 1967; Ross & Newman 1976; Ross & Frick 2011). In light of these considerations, we pursued a thin section analysis of the inner parietal architecture of some extant and extinct coronuloids, as detailed in the following section.

## PARIETAL MICROSTRUCTURE

**Figure 5:**
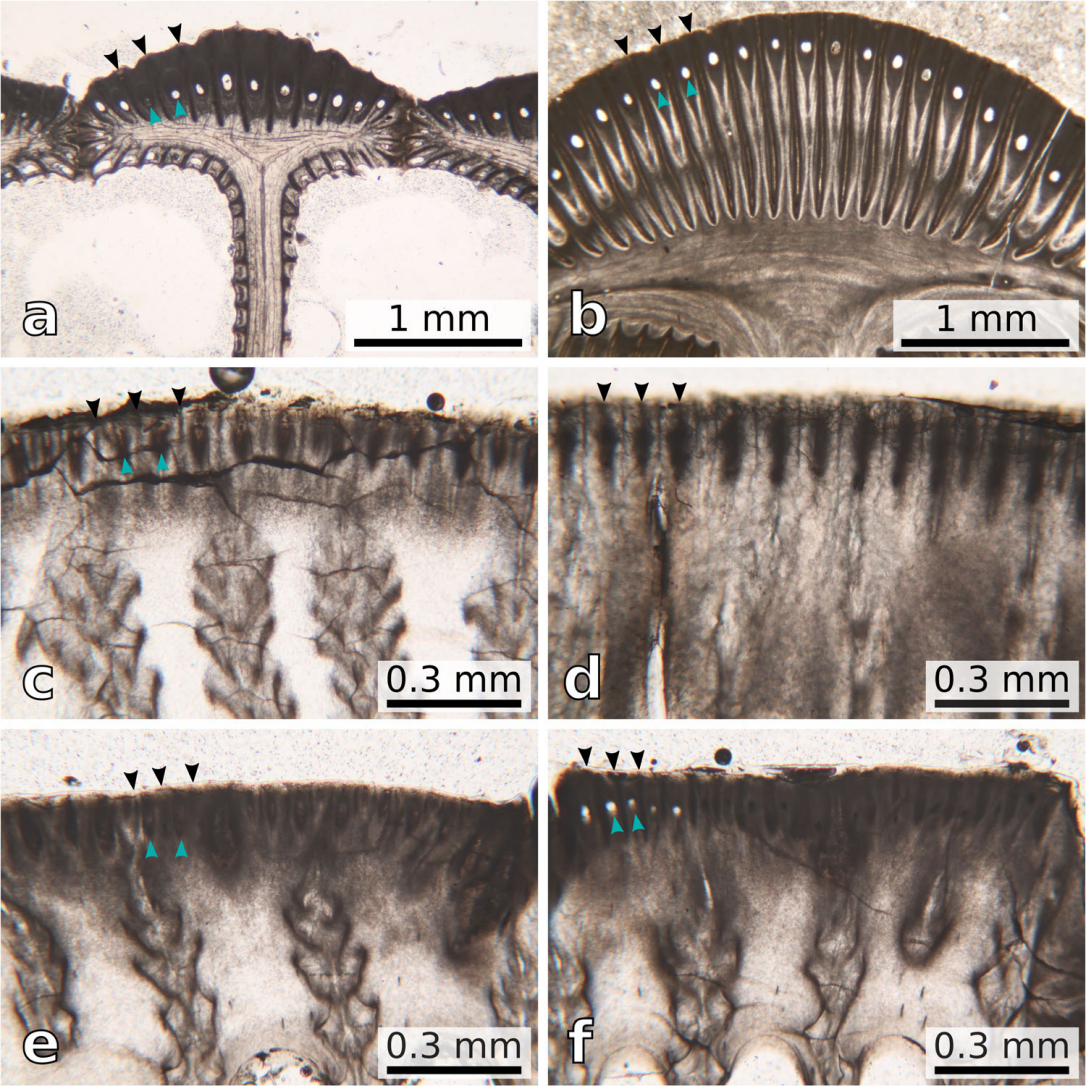
Comparison between the inner parietal microstructure of some extant and extinct coronuloids, with indication of the external longitudinal parietal canals (turquoise arrowheads) and septa (black arrowheads). The specimens are oriented so that their outer wall faces upwards in all the six panels. (a) *Coronula diadema*, thin section through the secondary outer lamina—note the presence of minor longitudinal parietal septa and canals lining the exterior of the primary T‐shaped flanges. (b) †*Coronula bifida*, thin section through the secondary outer lamina—note the presence of minor longitudinal parietal septa and canals lining the exterior of the primary T‐shaped flanges. (c) *Chelonibia caretta*, thin section through the paries—note the presence of external longitudinal parietal canals and septa; the former are secondarily filled by an amber‐colored shelly material. (d) *Chelonibia testudinaria* (*testudinaria* morph), thin section through the paries—note the absence of external longitudinal parietal canals; the external longitudinal parietal septa are still identifiable, but they are appressed to each other, so that no interseptal space is created in‐between them. (e) *Chelonibia testudinaria* (*patula* morph), thin section through the paries—note the presence of external longitudinal parietal canals and septa; the former are secondarily filled by a brownish shelly material. (f) *Chelonibia testudinaria* (*patula* morph), thin section through the paries—note the presence of external longitudinal parietal canals and septa; the former are partly secondarily filled by a dark (presumably carbonaceous) material, whereas five of them are still void. All the photomicrographs were taken in plane‐polarized light. For a general indication of which parts of the shells are being illustrated, please refer to Fig. 2f,h (for what regards panels (a) and (b)) and Fig. 2e,g (for what regards panels (c), (d), (e), and (f).

As highlighted elsewhere (e.g. Bianucci *et al*. 2006; Ross & Frick 2011; Kim *et al*. 2020), the shell of the extant whale barnacle *Coronula diadema* is characterized by the presence of macroscopic T‐shaped flanges (i.e. the parietal ribs or buttresses; Davis 1972) that abut from the outer wall and coalesce distally to form a secondary outer lamina. Microscopic, secondary T‐shaped flanges, similar to those of †*Chelonibia zanzibarensis* and *Platylepas* spp., can also be observed lining the primary (i.e. macroscopic) T‐shaped flanges, both inside and outside the secondary outer lamina (Fig. 5a). This character was first observed by Darwin (1854: p. 404), who reported on minute canals occurring on the outside of the compartment parietes of extant *Coronula* shells. Darwin (1854) described these canals as filled by the barnacle's corium. Pilsbry also noted that, in many coronulids, “there are pores in the outer layer of the parietes” (Pilsbry 1916: p. 269). According to Pilsbry (1916), these pores “apparently were formed by the deepening and closing over of external striae”. Based on personal observations by two of us (AC and WAN) on specimens of *C. diadema*, we concur that the substance identified as the corium by Darwin (1854) might rather be the skin and blubber of the host whale. In *C. diadema*, the exterior of the compartment parietes thus features an alternation of external longitudinal parietal tubes, appearing in thin sections as rounded pores, and external longitudinal parietal septa terminating in T‐shaped flanges, appearing in thin sections as built around a dark‐colored central line that recalls the main axis (*sensu* Coletti *et al*. 2018b) of the interlaminate figures of several balanid species. A much similar structure is observed in the extinct whale barnacle species †*Coronula bifida* (Fig. 5b). Surprisingly, distinct external longitudinal parietal canals and related T‐shaped flanges could also be observed in *Chelonibia caretta* (Fig. 5c). In this living species, the external longitudinal parietal septa and canals are distinctly smaller than those observed in *Coronula* spp. and †*C. zanzibarensis*, and their spatial frequency is consequently higher. The external longitudinal parietal canals of *C. caretta* are delimited by T‐shaped (or Y‐shaped) flanges, and they are invariantly secondarily filled by an amber‐colored shelly material (Fig. 5c). In the extant *Chelonibia testudinaria* (*testudinaria* morph), obvious external longitudinal parietal canals are absent; however, the external longitudinal parietal septa are still identifiable, but they are appressed to each other, so that no interseptal space is created in‐between them (Fig. 5d). Such a peculiar arrangement of the external longitudinal parietal septa was previously illustrated by Davis (1972: fig. 8), who also described the outer wall of *C. testudinaria* (*testudinaria* morph) as comprising “elongate vertical blocks of calcium carbonate”. *Chelonibia testudinaria* (*patula* morph) exhibits small‐sized external longitudinal parietal canals and septa that strongly recall those of *C. caretta*, the former being similarly secondarily filled by a brownish shelly material (Fig. 5e). Locally, however, the external longitudinal parietal canals of *C. testudinaria* (*patula* morph) are still void or secondarily filled by a dark, presumably organic material (Fig. 5f).

For summarizing, in agreement with Darwin (1854) and Pilsbry (1916), external longitudinal parietal canals were observed lining the secondary outer lamina of *Coronula* spp. (Fig. 5a,b). Similar yet distinctly finer tubes were also observed in the extant chelonibiids *C. caretta* and *C. testudinaria* (*patula* morph) (Fig. 5c,e,f). To our knowledge, this is the first report of external longitudinal parietal canals—a character thought to be exclusive of Coronulidae and Platylepadidae (e.g. Ross & Frick 2011)—in chelonibiids. Whereas the external longitudinal parietal canals of *C. caretta* are invariantly filled by calcareous material (Fig. 5c), they are sometimes void or filled by some sort of dark material in *C. testudinaria* (*patula* morph) (Fig. 5f).

## DISCUSSION

### Coronuloid monophyly

While regarding the turtle and whale barnacles as a “very natural group,” Darwin (1854) could not find soft‐ or hard‐part characters to substantiate this intuition. Darwin (1854) was thus led to discharge Leach's and Gray's subfamily Coronulinae, which was first proposed (under the name “Coronulidea”, intended as a new family in the order Acamptosomata) by Leach (1817) to embrace all the genera of epizoic acorn barnacles known thus far (i.e. *Tubicinella* Lamarck, 1802, *Coronula* and *Chelonibia*). Recent studies focused on the systematics and taxonomy of the so‐called turtle and whale barnacles (e.g. Newman 1996; Ross & Frick 2007, 2011; Harzhauser *et al*. 2011; Hayashi 2013; Collareta & Newman 2020) have generally accepted the existence of a monophyletic group (currently referred to as the superfamily Coronuloidea) that encompasses the chelonibiids, coronulids, and platylepadids, as well as the enigmatic Eocene form †*Emersonius cybosyrinx* Ross in Ross & Newman, 1967. The molecular phylogenetic analyses by Hayashi *et al*. (2013) and Pérez‐Losada *et al*. (2014) have lent support to this interpretation by demonstrating that the 3 living families of Coronuloidea do indeed comprise a monophyletic group. Nevertheless, as already mentioned, morphological characters that might strengthen the hypothesis of a common origin of the modern turtle and whale barnacles have long been missing.

Here, we argue that the presence of external longitudinal parietal canals defined in‐between adjoining T‐shaped flanges that abut from the outer lamina of the shell (Figs 2g,h, 4a, and 5a–c,e,f) is a character that can be found in members of all the three living families of Coronuloidea. Microscopic secondary T‐shaped flanges, and the related external longitudinal parietal canals, are present in several species of the type genus of Platylepadidae, *Platylepas* (e.g. *Platylepas coriacea* Monroe & Limpus, 1979, *Platylepas hexastylos*, and †*Platylepas mediterranea*), where they occur together with more conspicuous parietal bolsters (*sensu* Davis 1972) such as large internal mid‐ribs (Ross & Frick 2011). Macroscopic (i.e. primary) T‐shaped flanges, appearing as parietal buttresses whose distal terminations coalesce to form a secondary outer lamina (Fig. 2), represent the most striking character of the shell of the coronuloid genera *Coronula* and *Cetopirus*, but microscopic (i.e. secondary) T‐shaped flanges are also present in both genera (lining the external surface of the secondary outer lamina; Darwin 1854; Pilsbry 1916; this work). Microscopic T‐shaped flanges also occur in *Tubicinella* (which was regarded as a likely independent entry into the guild of whale barnacles by Seilacher 2005) and *Cryptolepas* Dall, 1872, two genera in which the larger primary T‐shaped flanges are not present (Pilsbry 1916; Davadie 1963; Ross & Frick 2011; Bosselaers & Collareta 2016). These structures strongly recall similar features observed in *Platylepas* (Davadie 1963) and *Chelolepas* Ross and Frick, 2007 (personal observation by AC). Finally, the present study demonstrates that the extant chelonibiid genus *Chelonibia*, whose shell has long been regarded as solid, displays more or less developed external longitudinal parietal canals that are separated from each other by external longitudinal parietal septa terminating in microscopic T‐shaped flanges (Figs 2g and 5c,e,f). As this kind of structure is not known outside Coronuloidea, we propose that the presence of external longitudinal parietal canals represents an autapomorphy of this superfamily.

Coupled with the strongly reduced opercula, the observation of external longitudinal parietal canals thus represents one of the most discriminating characters of Coronuloidea, although one subject to reversal in various genera and species (see subsection below).

### Functional morphology

While Darwin (1854) described the T‐shaped flanges in the platylepadids and whale barnacles, he seemingly equivocated over them in the chelonibiids. However, Davis (1972) produced good images of what he described as elongated vertical blocks of calcium carbonate comprising the outer wall of *Chelonibia testudinaria* and we demonstrated the occurrence of unambiguous T‐shaped flanges in the new species from the Miocene of Zanzibar. That said, what is the function of these peculiar structures of the coronuloid outer wall?

In the extant *Cetopirus* Ranzani, 1817 and *Coronula*, the primary T‐shaped flanges are essentially cutting devices that allow for coring prongs out of the whale skin, thus favoring the anchorage of the barnacle to its molting substrate (Seilacher 2005; Kim *et al*. 2020). This is demonstrated by the observation that the cavities that develop in‐between them are regularly filled by shreds of solid whale skin, well above the surrounding skin surface (Darwin 1854; Seilacher 2005). The microscopic T‐shaped flanges of *Cryptolepas* might have a similar function (Seilacher 2005), and those of *Coronula*, *Cetopirus*, and *Tubicinella* are also interpretable as tools that allow to grasp and penetrate into the substrate by cutting out thin strings of the host’ skin. In the smaller‐sized platylepadid species *P. hexastylos*, similar yet very thin longitudinal canals and septa are also observed on the exterior of the compartment parietes. These structures are also functional as substrate graspers that favor the fouling of the relatively ductile turtle skin.

In the *patula* morph of *Chelonibia testudinaria*, the external longitudinal parietal tubes are very thin and mostly filled by an amber‐colored shelly material. It should be noted that this living form mostly adheres superficially onto hard substrates such as the exoskeleton of crabs and horseshoe crabs (e.g. Pilsbry 1916; Stubbings 1967; Ross & Jackson 1972; Jones *et al*. 2000); it rarely occurs on the carapace of sea turtles (Kitsos *et al*. 2005) and has even been collected from submerged defleshed bones (Frazier & Margaritoulis 1990; Collareta & Bianucci 2021). It is reasonable to suppose that the T‐shaped flanges of *C. testudinaria* (*patula* morph) are largely no longer functional, their obliteration by secondary biogenic carbonate being likely contemporaneous with the peripheral growth of the mural plates. In *Chelonibia caretta*, a relatively deep‐penetrating species that inhabits the carapace and plastron of sea turtles, the external longitudinal parietal tubes are also very thin and completely calcified secondarily. It is likely that the substrates inhabited by *C. caretta* are not ductile enough to be string‐sliced by the minute, closely spaced T‐shaped flanges of chelonibiids. As a consequence of that, the shells of *C. caretta* often display invaginations of the periphery that allow for encapsulating larger portions of the host's keratinous scutes (e.g. Monroe 1981).

In turn, the large‐sized T‐shaped flanges of †*Chelonibia zanzibarensis* were, in our opinion, much likely functional. They correlate with obvious riblets in the lower portion of the compartment parietes and define broad tubes in‐between them (Fig. 4b,c). The latter, where locally revealed by sub‐apical erosion of the shell (Fig. 4a), appears as partially filled by a dark, likely carbonaceous material that might represent the remainder of the host's integument. The truncated‐conical shell shape of †*C. zanzibarensis* compares favorably with the overall aspect of specimens of *Platylepas* for which penetration into a soft substratum has been observed (e.g. Pilsbry 1916) or presumed (Collareta *et al*. 2019). Indeed, as illustrated by the presence of a distinct substrate imprint on the shell exterior, ≈5 mm above the shell base (Fig. 4b), the lowermost portion of the holotype specimen was likely embedded within the substrate. It is thus reasonable to hypothesize that the relatively large‐sized T‐shaped flanges of †*C. zanzibarensis* were actively involved, as substrate graspers, in keeping the barnacle attached to its host.

As detailed above, whereas miniature T‐shaped flanges and related external longitudinal parietal canals are present in both *Chelonibia caretta* and the *patula* morph of *Chelonibia testudinaria*, neither the former nor the latter exploit the aforementioned structures as substrate graspers, the external longitudinal parietal canals of these forms being largely secondarily filled by biogenic calcite (Fig. 4c,e). These structures are most parsimoniously interpretable as not functional and more precisely as vestigial, that is, functional versions of them were likely present in some common ancestor of the living chelonibiids. †*Chelonibia zanzibarensis* is consistent with such a putative ancestral form in terms of shell architecture, and consequently, it represents a key taxon for reconstructing how and from where the extant chelonibiids evolved. The retention of vestigial T‐shaped flanges and external longitudinal parietal canals in *C. caretta*, a species that shares several shell traits with †*C. zanzibarensis*, is thus somewhat expectable; in turn, it comes as some surprise in the *patula* morph of *C. testudinaria*. Indeed, the latter is regarded as a conspecific of *C. testudinaria* (*testudinaria* morph), in which neither the T‐shaped flanges nor the external longitudinal parietal canals could be recognized, its external longitudinal parietal septa being appressed to each other so that no interseptal space is created in between them (Fig. 4d). More in general, the origin of the high degree of polymorphism of the extant species *C. testudinaria* is an outstanding open question that definitively merits to be properly addressed. Some considerations and perspectives on this prime issue are provided in the last section of the present paper.

**Figure 6:**
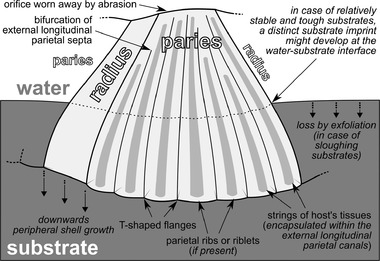
Schematic diagram of the substrate grasping mechanism of a generalized early coronuloid (here exemplified by a carinolateral compartment) provided with functional T‐shaped flanges and external longitudinal parietal canals.

Here, we argue that the development of T‐shaped flanges and external longitudinal parietal canals represent the key hard‐part modification that enabled early coronuloids to effectively grasp (and spread on) an amazingly diverse spectrum of sloughing, more or less penetrable substrates, including the marine mammal skin (Fig. 6). In the present work, similar structures have been detected in one of the oldest ascertained member of the crown Coronuloidea, that is, †*C. zanzibarensis*, and this demonstrates their occurrence in members of all the 3 living coronuloid families. Though many extant coronuloids still exploit the T‐shaped flanges and external longitudinal parietal canals to enhance anchorage to their host, these structures are vestigial if not hardly discernible in extant chelonibiids, and might even have secondarily disappeared in some coronulids and platylepadids. This could be the case with *Xenobalanus globicipitis* Steenstrup, 1852, in which the shell is miniaturized and deeply entombed within the host's skin (Seilacher 2005), and something similar could be said for *Stephanolepas muricata* Fischer, 1886 (Frick *et al*. 2011). In these species, the deep‐penetrating shell appears as everted and sends external projections such as spines, knobs, and imbricating scales that enhance anchorage (Seilacher 2005; Frick *et al*. 2010), but chemical mediation might also have a role in favoring attachment to the living substrate (Frick *et al*. 2011). That said, further close inspections of these highly derived, seemingly solid‐walled coronuloids are needed to better elucidate their attachment strategies.

### Inferences on the host habits of †*Chelonibia zanzibarensis*

What kind of living substrate did the holotype of †*C. zanzibarensis* attach to? Considering the verified hosts of extant coronuloids, we might discard the invertebrates (i.e. crabs, horseshoe crabs and molluscs), because their hard, thin exoskeletons would not allow for substantial penetration by the barnacle shell. All the groups of vertebrate hosts of extant coronuloids (i.e. sea turtles, cetaceans, sea cows, crocodylians, sea snakes, and gars) were already present in early‐middle Miocene times. Given the large‐sized external longitudinal parietal canals of †*C. zanzibarensis*, the kind of substrate inhabited by this extinct chelonibiid should have been somewhat penetrable and ductile to allow for coring and string‐slicing by the T‐shaped flanges. At the same time, it should have been tough and stable enough to account for the observation of a distinct mark on the compartment parietes. Not least, the flat calcareous surface formed by the radiating internal parietal septa of the holotype of †*C. zanzibarensis* suggests that the shell base might have spread over an impenetrable, rather planate structure. It must be noted that, during the early and middle Miocene, whale barnacles had likely still not evolved, their geologically oldest fossils being as young as the late Miocene (Buckeridge *et al*. 2019). Therefore, in absence of more specialized forms, there would have been the opportunity for chelonibiids to experiment settlement on the skin of cetaceans (see also Collareta *et al*. 2016 in this respect). That said, the marine mammal skin might be too prone to exfoliation and compliant to the coring activity of barnacles for being in any way responsible for the formation of a distinct substrate imprint. One such substrate might be provided by the carapace of the leatherback turtles (Chelonii: Dermochelyidae), which lacks the horny scutes of cheloniids, being instead comprising a mosaic of dermal elements (the so‐called “ossicles”) embedded in a layer of tough, rubbery skin (Pritchard 1971; Delfino *et al*. 2013). In early and middle Miocene times, dermochelyids were widespread and abundant with the genera †*Psephophorus* Meyer, 1847 and, most likely, †*Natemys* Wood *et al*., 1996 (Delfino *et al*. 2013; Bianucci *et al*. 2018; Peters *et al*. 2019). In light of this hypothesis, the flat calcareous surface formed by the internal longitudinal parietal septa of †*C. zanzibarensis* might be interpreted as resulting from the contact between the barnacle shell and the “epithecal” mosaic of dermal elements of the carapace of a leatherback turtle.

### Genetics, ontogeny, atavism, and the origin of polymorphism in *Chelonibia testudinaria*

The genetic analyses by Cheang *et al*. (2013) and Zardus *et al*. (2014) demonstrated that *C. testudinaria* (occurring on marine turtles) and the generally smaller species *Chelonibia patula* (occurring on a variety of largely arthropod and gastropod hosts) and *Chelonibia manati* (occurring on manatees) are all the same species. This assemblage of previously described “species”, generally occupying different hosts, readily qualifies them as morphs, since there is no doubt they comprise the same species‐level taxon and they are so classified herein along with other extant nominal species of *manati*‐ and *patula*‐like forms such as *Chelonibia ramosa* Korschelt, 1933 (Table 1). Of all the species that have thus been synonymized, the first to be named is *C. testudinaria*, whose formal description dates back to Linnaeus (1758), which might make it appear more important than for example, *C. patula*, whose formal description dates back to Ranzani (1817). However, it must be noted that the older publication date of the former is by no means a mark of ecological or phylogenetic prominence among its peers.

The observation that *C. patula* and *C. testudinaria* are actually different morphs of the same species rather than different species seemingly invalidates the supposition that the former, which exploits less recently appeared host types such as crustaceans and mollusks as well as inanimate substrates, is the more primitive (i.e. generalized and archaic‐looking; Frazier & Margaritoulis 1990) of the coronuloids (see also Hayashi *et al*. 2013 in this respect). *Chelonibia patula* was indeed placed at the bottom rather than at the top of the phylogenies proposed by Ross and Newman (1967) and Monroe (1981). The basis for it being considered primitive by earlier workers relies in the following suite of characters: wall relatively thin; tripartite rostral complex having fully visible sutures and whose constituting plates are largely separable in smaller (i.e. younger) individuals at least; radii conspicuous the rest of the way around; and sheath weakly developed, the spaces between the internal longitudinal parietal septa being not secondarily filled with skeletal material. Then too, the *patula* morph of *C. testudinaria* is a generalist occurring on a wide variety of hosts compared to the more heavily built and streamlined *testudinaria* form that is primarily found on sea turtles, where when on the carapace it can even move forward to more favorable positions near its host's head (Moriarty *et al*. 2008).

Thus, we have an enigma, namely, an epizoic barnacle species (*C. testudinaria*) comprising a large, rather heavily built, host‐specialized form (the *testudinaria* morph) as well as smaller, lightly built forms, including a generalist hosted largely by gastropods and various marine arthropods (the *patula* morph): these are 2 very distinct morphs of the same species, and both have apparently existed since the late Neogene (Collareta 2020, and references therein). So it behooves us to look at both paleontology and ontogeny to see what insights they may provide on the origin of these quite differently structured forms, adapted to so many different hard‐shelled hosts, most of whom slough their surfaces and/or periodically molt.

The fossil record of the subfamily †Protochelonibiinae includes the Oligocene, relatively thin‐shelled †*Protochelonibia mellleni* (Zullo, 1982), the geologically oldest member of Chelonibiidae, which instructively could produce a lobate basal margin much as the *manati* morph of *C. testudinaria* can today (Zullo 1982), whereas the remarkably gregarious †*Protochelonibia submersa* Harzhauser & Newman in Harzhauser *et al*., 2011 and †*Protochelonibia capellinii* (De Alessandri, 1895) thrived in the Miocene and Pliocene, respectively. The protochelonibiines did not persist into the Quaternary but we do not know what their hosts were up to then. The fossil record of the extant subfamily Chelonibiinae appears to date back to the Miocene; it includes both lightly and heavily built forms such as the *testudinaria* and *patula* morphs of *C. testudinaria*, as well as †*Chelonibia zanzibarensis* and †*Chelonibia solida*. The shell architecture of the latter might fit within the variability of *C. testudinaria* (Ross 1963), so that †*C. solida* could comprise another morph of this extant species (as originally proposed by Withers 1929); however, this would mean extending back the stratigraphic range of *C. testudinaria* for more than 10 million years in absence of an unambiguous fossil record. As regards †*C. zanzibarensis*, while it is an ally of *C. caretta* rather than of *C. testudinaria*, there is no way of telling whether or not it was a member of a cluster of morphs like the latter. Therefore, while the early forms of chelonibiids in the subfamily †Protochelonibiinae were light in structure, a few heavily built forms of Chelonibiinae have been present since the Miocene. Besides demonstrating that modern‐looking members of *C. testudinaria* have been present from the late Neogene at least (Collareta *et al*. 2016; Collareta 2020), paleontology does not shed much further light on the very origin of this complex extant species.

In a recent paper on *C. testudinaria*, Cheang *et al*. (2013) went beyond than calling the diversity of its forms simply morphs or ecotypes; rather, they suggested that a number of environmental stimuli are likely responsible for what a cyprid larva of any one of the morphs might select to settle on. While the larval stages (6 naupliar and 1 cyprid stage) have been well described (Zardus & Hadfield 2004), the juvenile ontogeny of *Chelonibia per se* has not been reported on. Yet it can be said with reasonable confidence, since settlement is on firm surfaces rather than penetrating a host's fleshy tissue such as a coral or sponge, that the pre‐settlement, post‐larval metamorphic stages are likely comparable to the metamorphic stage that first settles in other balanomorphs. It would thus be during the early ontogeny, following cementation and settlement, that one might expect to find adaptations in shell structure relative to the substratum in what would be a relatively fragile shell. We now know this early shell would differ from those of all other balanomorphs except other coronuloids in having external longitudinal parietal septa developing unique tissue‐grasping structures deployed around the outer basal margin in the form of T‐shaped flanges encompassing any host tissue they can grasp and tightening their grip as the shell grows by closing the tops of the T‐shaped flanges, creating the secondary outer lamina in the process.

It is at this point in ontogeny that “choices” (activation and/or deactivation of certain genes) in response to various environmental stimuli need to be made by the juvenile (see also Cheang *et al*. 2013). If it were not for the genetics, one might be looking to atavism (Tomić & Meyer‐Rochow 2011) as the source of a new form, especially since there is a nuance of an appropriate atavist in early juveniles of the *testudinaria* morph of *C. testudinaria*. However, it is the environmental aspect of this hypothesis that can be subjected to experimental validation or falsification and therefore it would surely be worth looking into (Sloan *et al*. 2014). For example, something as simple as adding a swarm of cyprid larvae of the *testudinaria* morph of *C. testudinaria* to an isolated tank with an appropriate swimming crab, xiphosuran or gastropod, but no turtle to see if one gets the *patula* morph of *C. testudinaria* might suffice. Once one has observed settlement, the tank should probably get fresh circulating seawater in order to feed the developing barnacles to maturity.

## COMPETING INTERESTS

The authors declare that they have no competing interests.
